# The Impact of Polycystic Ovary Syndrome on Mood Disorders: A Cross-Sectional Questionnaire Study on Chronotypes, Social Jetlag, and Night Eating Habits

**DOI:** 10.3390/jcm14197068

**Published:** 2025-10-07

**Authors:** Senol Senturk, Mehmet Kagitci, Meltem Pusuroglu, Ugur Avci, Tahsin Gokhan Telatar, Bahar Kefeli Col, Nalan Kuruca, Deniz Dereci Delibas, Safak Hatirnaz, Filiz Mercantepe, Andrea Tinelli

**Affiliations:** 1 Department of Obstetrics and Gynecology, Faculty of Medicine, Recep Tayyip Erdogan University, Rize 53350, Türkiye; mehmetkagitci1@hotmail.com (M.K.); dr.aates.9@gmail.com (N.K.); denizdereci0@gmail.com (D.D.D.); 2 Department of Psychiatry, Faculty of Medicine, Recep Tayyip Erdogan University, Rize 53350, Türkiye; meltempusuroglu@gmail.com; 3 Department of Endocrinology and Metabolism, Faculty of Medicine, Recep Tayyip Erdogan University, Rize 53350, Türkiye; ugur.avci@erdogan.edu.tr (U.A.); filiz.mercantepe@saglik.gov.tr (F.M.); 4 Department of Public Health, Faculty of Medicine, Recep Tayyip Erdogan University, Rize 53350, Türkiye; tahsingokhan.telatar@erdogan.edu.tr; 5 Güneysu Vocational School of Physical Therapy and Rehabilitation, Recep Tayyip Erdogan University, Rize 53350, Türkiye; bahar_kefeli@hotmail.com; 6 Mediliv Medical Center, Department of Obstetrics and Gynecology, Samsun 55100, Türkiye; safakhatirnaz@gmail.com; 7 Department of Obstetrics and Gynecology, Cericsal (Centro di RIcerca Clinico Salentino), “Veris delli Ponti Hospital”, Via Giuseppina Delli Ponti, 73020 Scorrano, Italy; andreatinelli@gmail.com

**Keywords:** PCOS, questionnaire, depression, anxiety, chronotype, social jetlag, eating habits

## Abstract

**Objective**: To determine the prevalence of mood and eating disorders, chronotype, and social jetlag in a cohort of women with polycystic ovarian syndrome (PCOS). **Methods**: A total of 70 patients, 35 with PCOS and 35 healthy controls, aged between 18 and 40 years, were included in the study. PCOS was diagnosed according to the Rotterdam criteria. Five different questionnaires, namely the “Morningness–Eveningness Questionnaire (MEQ)”, “Social Jetlag Status (SJL)”, “Night Eating Questionnaire (NEQ)”, “Beck Depression Inventory (BDI)”, and “Beck Anxiety Inventory (BAI)”, were administered to patients with and without PCOS, and the “total questionnaire scores” of both groups were compared. **Results**: In addition to BMI (*p* = 0.004), serum insulin (*p* < 0.001), HOMAIR (*p* < 0.001), total testosterone (*p* = 0.006), DHEAS (*p* = 0.004), and LH (*p* < 0.001) levels were significantly higher in women with PCOS than in the controls. BAI (*p* = 0.006), BDI (*p* = 0.007), and NEQ (*p* = 0.013) scores of participants with PCOS were significantly higher than those in the control group, while MEQ scores were significantly lower than those in the control group (*p* = 0.005). When categorized according to the total test scores, the number of individuals with moderate and severe anxiety was significantly higher in the PCOS group than in the control group (*p* = 0.030). Morningness was significantly lower in the PCOS group than in the control group, whereas eveningness was higher than that in the control group (*p* = 0.013). There was no difference between the PCOS and control groups in terms of the number of individuals with SJL ≥ 2 h and night eating disorders. The NEQ score was positively correlated with BAI, BMI, insulin, and HOMA-IR. Both the BDI and BAI scores were positively correlated with BMI, HOMA-IR, and total testosterone levels. **Conclusions**: PCOS can lead to mood, appetite, and circadian rhythm issues through variations in chronotype. PCOS-related endocrine, metabolic, and adiposity factors influence mood, eating habits, and chronotype disorders.

## 1. Introduction

Polycystic ovary syndrome (PCOS), the most common endocrine pathology detected in the reproductive age group, impairs the quality of life of individuals affected both physiologically and psychologically due to the accompanying hormonal, metabolic, dermatological, and adiposity pathologies [1]. Metabolic pathologies caused by lipid, carbohydrate, and hormonal pathway deterioration can lead to eating disorders, sleep disorders, and deterioration in mental health parameters [1,2]. Many patients with PCOS find it difficult to adhere to the prescribed dietary adjustments, lifestyle modifications, pharmaceutical and natural supplements, and increased physical activity [2]. PCOS significantly impacts the quality of life and psychological well-being of those affected by it. Obesity, negative body image, menstrual irregularities, and anxiety about not being able to conceive contribute to psychological distress in many patients with PCOS [3]. The 2023 ESHRE guidelines state that moderate-to-severe anxiety and depression disorders are common in adults with PCOS and that screening for these disorders is necessary. In addition to psychological problems, patients with PCOS should be evaluated for self-harm and suicidal thoughts [4].

In addition to psychological problems, eating disorders and social isolation can be observed at varying intensities in PCOS phenotypes, especially those accompanied by obesity and hyperandrogenemia [5,6,7]. Circadian rhythm, determined by factors such as sex, age, comorbid endocrine and metabolic diseases, light exposure, eating behaviors, and social timing [8], is a behavioral process that repeats every 24 h [9]. As a result of increased exposure to artificial light due to changing working hours and rapid and continuous travel [10], the concept of “circadian mismatch” has emerged [11,12]. Circadian mismatch, which is especially common in shift work, causes short and ineffective sleep durations and wakefulness periods [13,14,15]. Social jetlag (SJL) is a circadian disorder resulting from an imbalance between sleep/wake periods on school/workdays and holidays [16]. SJL is defined as the average difference between sleep duration on workdays and sleep duration on holidays [16]. Chronotype, which is closely related to circadian rhythm, defines the periods in which an individual tends to be awake or asleep [8]. Most individuals have innate sleep and wakefulness periods [17]. Accordingly, individuals exhibit three different chronotypes: “morning type (MCT),” “intermediate type (ICT),” and “evening type (ECT).” While MCT wake up early and begin their daily activities, ECT usually wake up late and perform most of their activities near and during the evening [15]. Eating disorders, common among adolescents and individuals of reproductive age, can lead to serious mental and mood disorders if left untreated. Eating disorders in young individuals with PCOS are often accompanied by depression and anxiety [8,18]. Data on the prevalence of mood swings, eating disorders, social jetlag, and chronotypes in individuals with PCOS are limited [5]. In a recent study, our team published a comprehensive study on the prevalence of mood and eating disorders, as well as social jetlag and chronotypes, in patients with primary dysmenorrhea [19]. Although nutritional and lifestyle changes, as well as psychological support, are often recommended as first-line treatments for individuals with PCOS [2,20], no comprehensive survey study has been conducted on whether PCOS affects the frequency of anxiety, depression, chronotype, eating disorders, and social jetlag. This survey study was designed to determine the prevalence of mood and eating disorders, chronotype, and social jetlag in a cohort of women with polycystic ovarian syndrome (PCOS). In this prospective case–control study, five different questionnaires, namely the “Morningness–Eveningness Questionnaire (MEQ),” “Social Jetlag Status (SJL),” “Night Eating Questionnaire (NEQ),” “Beck Depression Inventory (BDI),” and “Beck Anxiety Inventory (BAI),” were administered to patients with and without PCOS, and the total questionnaire scores of both groups were compared.

## 2. Materials and Methods

### 2.1. Patient Selection

Recep Tayyip Erdoğan University Training and Research Hospital Non-Interventional Clinical Research Ethics Committee approved this prospective survey study conducted on PCOS and non-PCOS controls at the Gynecology and Obstetrics Clinic (permission number: 2023/67, date: 9 March 2023). Following ethical approval, the survey was conducted between March 2023 and August 2023. PCOS was diagnosed according to the Rotterdam criteria for PCOS. Participants in the PCOS and control groups were matched for age and BMI. PCOS and control participants were selected from middle-income individuals with at least a high-school degree. Study and control group participants who satisfied the inclusion requirements based on the preliminary evaluation were chosen voluntarily to participate in this cross-sectional survey. The survey was initiated after the patients provided consent. During the study, five different anonymous multiple-choice surveys were administered to the participants in accordance with the “Helsinki Declaration” and the “Good Clinical Practice Guidelines.” Participants who did not answer any surveys completely were not included in the study; therefore, there was no missing data.

G* Power 3.1.9.7 was used to determine sample size. With an effect size of Cohen’s d = 0.80, calculated using the *t*-test for independent groups, and a statistical power of 90% at a significance level of α = 0.05, the minimum required total sample size was determined to be 68 (34 participants per group).

According to the power analysis, 70 patients (35 with PCOS and 35 healthy controls without PCOS), aged between 18 and 40 years, were included in the survey. PCOS was diagnosed according to the Rotterdam criteria. The participants in the PCOS and control groups were matched for age and BMI. Women who met at least two of the criteria for hyperandrogenemia, ovulatory dysfunction, and polycystic ovarian morphology, as determined by the European Society for Human Reproduction and Embryology/American Society for Reproductive Medicine (ESHRE/ASRM), were diagnosed with PCOS [21]. Oligomenorrhea was defined as having less than six menstrual periods per year in previous years, while the presence of ≥12 follicles per ovary with a diameter of 2–9 mm and/or an increase in ovarian volume (>10 mL) was defined as polycystic morphology. Hyperandrogenism was defined clinically by hirsutism and/or acne and biochemically by total testosterone and free androgen index levels. Participants in the control group were selected from middle-income individuals with at least a high school education and no other endocrinopathy, including PCOS. Some of the controls were university students, while others were outpatients, and their ages and BMI were similar to those of the PCOS group. Unlike the PCOS group, some of the controls worked shifts. Of the 35 patients in the control group, 13 presented with menstrual irregularities, 8 with dysmenorrhea, 7 with acne and hair loss, and the remaining 7 with vaginal infection. After being provided with detailed information about the study, they voluntarily completed the survey. After the participants were explained in detail how to fill out the questionnaires, the filling process began. Luteinizing hormone (LH), follicular-stimulating hormone (FSH), dehydroepiandrosterone sulfate (DHES), insulin, and total testosterone levels, as well as other biochemical parameters, were measured in blood samples collected after an overnight fast of at least 8–10 h. The homeostasis model assessment of insulin resistance (HOMA-IR) was calculated using the following formula: insulin (μU/mL) × glucose (mg/dL)/405 [22].

### 2.2. Inclusion and Exclusion Criteria

The inclusion criteria were as follows: no history of pregnancy; no use of hormonal or antidepressant medication in the last six months; no endocrine disease other than PCOS; not working in a shift job; having at least a high school degree; and normal uterine anatomy. The exclusion criteria were as follows: history of pelvic inflammatory illness; history of prior pregnancy; reproductive organ pathology; endocrine disease other than PCOS; use of hormonal or antidepressant medications within the previous six months; working a shift job; and habitual use of cigarettes and alcohol. 

### 2.3. Instruments, Measurements, and Data Processing

After filling out the personal information form, all participants were administered the Social Jetlag (SJL), Night Eating Questionnaire (NEQ), Beck Depression Inventory (BDI), Beck Anxiety Inventory (BAI), and Morning and Evening Eating Questionnaire (MEQ). All participants who agreed to participate in the survey were provided with detailed information about the survey and how to answer it. They were asked to complete each survey in an anonymous, well-lit room without any time constraints. Participants whose personal data or survey responses were found to be incomplete in the first evaluation after the surveys were excluded from the study design. The Turkish-specific versions of each questionnaire used in this study were validated in previous local studies [15,16,17,18,19].

#### 2.3.1. MEQ

The chronotype determination questionnaire, initially developed by Horne and Ostberg [23] and subsequently evaluated for reliability in its Turkish adaptation by Punduk et al. [24], is a Likert-type scale comprising 19 questions. Each question had four possible responses. Participants received scores based on their selected answers, with questions 3, 4, 5, 6, 7, 8, 9, 13, 14, 15, and 16 scored from 1 to 4. Questions 1, 2, 10, 17, and 18 were scored from 1 to 5, whereas questions 11 and 19 were scored from 0 to 6. Question 12 was scored from 0 to 5. The scoring system ranges from 16 to 86, with low scores indicating an evening chronotype and high scores indicating a morning chronotype. At the end of the test, three different circadian types were classified as morning type (MCT) for those who scored between 59 and 86, intermediate type (ICT) for those who scored between 42 and 58, and evening type (ECT) for those who scored between 16 and 41.

#### 2.3.2. NEQ

The NEQ is a 16-question questionnaire originally developed by Allison et al. [25] and validated in Turkish by Atasoy et al. [26]. All participants were required to complete the initial nine questions. Questions 10–12 were designated for participants who experienced nocturnal awakenings, whereas questions 13 and 14 were intended for those who snacked during the night. All questionnaire items, except item 7, were assessed on a five-point Likert scale and scored between 0 and 4. The seventh question asked about mood changes during the day, with a score of 0 assigned to those who reported no changes. Questions 1, 4, and 14 were scored in reverse. The total score range is 0–52. Scores of 25 points or above were considered night eating syndrome (NES).

#### 2.3.3. SJL

The SJL refers to the difference between sleep patterns on workdays and holidays and provides information on the degree of circadian mismatch [10]. Participants were grouped as SJL < 2 h and SJL ≥ 2 h according to the difference between the median values of weekday and weekend sleep hours [19].

#### 2.3.4. BDI

The BDI is a questionnaire consisting of 21 questions that assesses depressive symptoms, the validity of which was established by Hisli [27]. The severity of symptoms was assessed by assigning scores ranging from 0 (not bothered at all) to 3 (severely bothered) for each response to the question. In line with previous studies, in the present study, those who scored 17 or more on the BDI were considered to have depression (+).

#### 2.3.5. BAI

The BAI is a 21-item self-report questionnaire that lists physical and psychological anxiety symptoms experienced in the previous month. Its validity in Türkiye was determined by Ulusoy et al. [28]. Participants rated how much each symptom bothered them in the past month, from 0 (not at all) to 3 (severe). According to the BAI scores, individuals were divided into three categories: those with mild anxiety (−8–15 points), those with moderate anxiety (−16–25 points), and those with severe anxiety (−26–63 points). Higher cumulative scores above these points indicate increased anxiety.

### 2.4. Statistical Analysis

Statistical analyses were performed using SPSS version 27 (IBM Corp., Armonk, NY, USA). Graphical representations were generated using GraphPad Prism 10.4.2 (GraphPad Software, San Diego, CA, USA). The Kolmogorov–Smirnov test was used to evaluate the normality of the data distribution. Data are presented as mean ± standard deviation or median (1st quartile–3rd quartile) for continuous variables and as frequency (percentage) for categorical variables, depending on the normality of the distribution. For the comparison of categorical data, the Chi-square test or Fisher’s exact test was applied. An independent samples *t*-test was used to compare continuous variables exhibiting a normal distribution, whereas the Mann–Whitney U test was employed for continuous variables that did not demonstrate a normal distribution. Spearman’s correlation analysis was conducted to ascertain the relationship between participants’ demographic, clinical, and laboratory data and questionnaire scores. A *p*-value of less than 0.05 was considered statistically significant for all statistical tests. R software (v. 4.3.3) and the “Metan” package were used to create a correlation heatmap [29].

## 3. Results

A total of 70 women, 35 with PCOS and 35 healthy controls, were included in this prospective case–control survey study. Participants in the control group were selected from individuals without any other endocrinopathy, including PCOS. The participants in the PCOS and control groups were matched for age.

**Figure 1:**
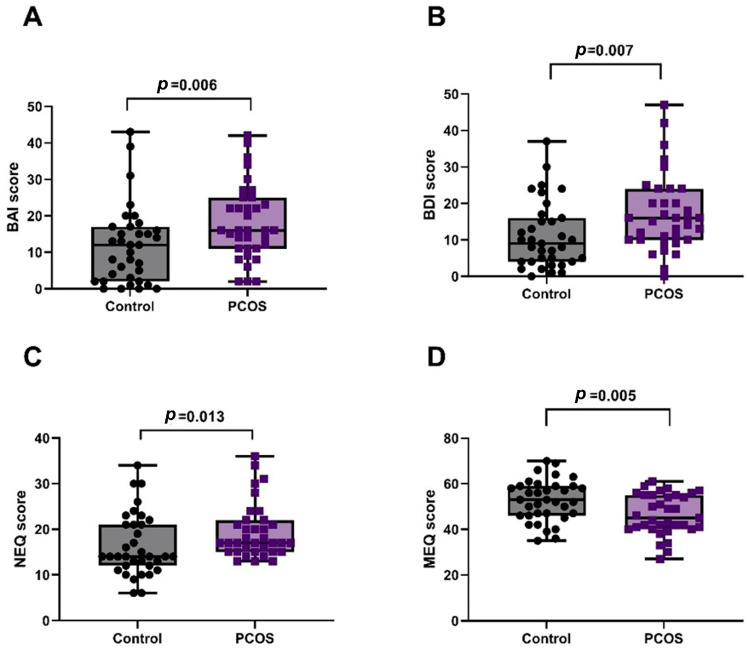
Comparison of questionnaire scores between PCOS and control groups. (**A**) BAI: Anxiety scores were significantly higher in PCOS (*p* = 0.006). (**B**) BDI: Depression scores were significantly higher in PCOS (*p* = 0.007). (**C**) NEQ: Night eating scores were significantly higher in PCOS (*p* = 0.013). (**D**) MEQ: Morningness–Eveningness scores were significantly lower in PCOS (*p* = 0.005).

The number of individuals with hirsutism in the PCOS group was significantly higher than that in the control group (*p* < 0.001). BMI (*p* = 0.004), insulin (*p* < 0.001), HOMA-IR (*p* < 0.001), LDL-C (*p* < 0.001), total cholesterol (*p* < 0.001), total testosterone (*p* = 0.006), DHEAS (*p* = 0.004), and LH (*p* < 0.001) levels were significantly higher in the PCOS group than in the control. There were no significant differences between the PCOS and control groups in terms of waist circumference, hip circumference, FBG, HDL-C, triglyceride, estradiol, FSH, progesterone, prolactin, TSH, and high-sensitivity C-reactive protein (hs-CRP) levels. As shown in Figure 1, the BAI (*p* = 0.006), BDI (*p* = 0.007), and NEQ (*p* = 0.013) scores of participants with PCOS were significantly higher than those in the control group, while the MEQ scores were significantly lower than those in the control group (*p* = 0.005). The demographic data of the participants in both groups are presented in detail in Table 1.

**Figure 2:**
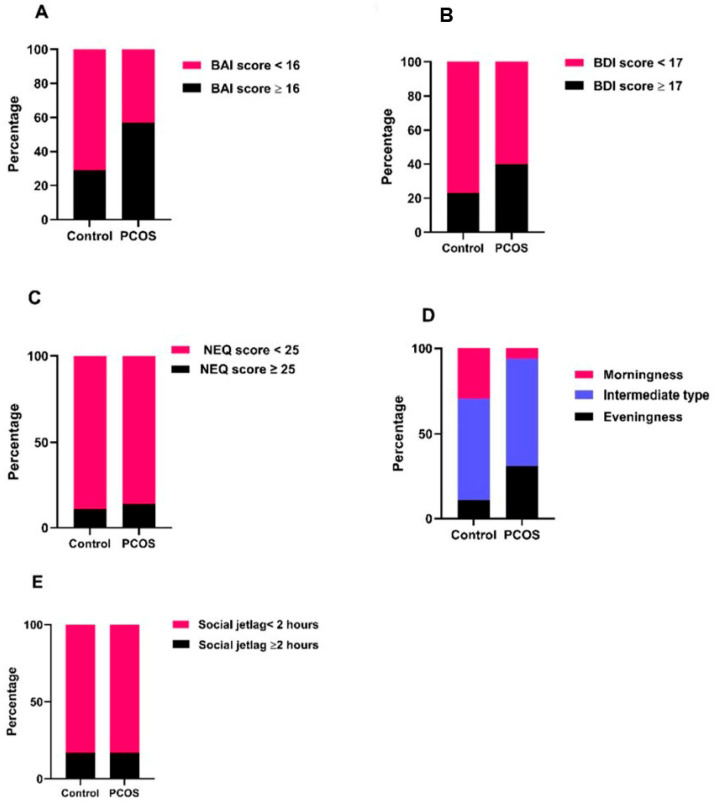
Graphical presentation of the distribution of pathological versus normal scores in both groups. (**A**) Beck Anxiety Inventory (BAI): The proportion of participants with moderate-to-severe anxiety (BAI ≥ 16) was significantly higher in the PCOS group compared to the control group (57.1% vs. 28.6%, *p* = 0.030). (**B**) Beck Depression Inventory (BDI): Although the number of participants with depression (BDI ≥ 17) was higher in the PCOS group (40.0%) than in the control group (22.9%), the difference did not reach statistical significance (*p* = 0.198). (**C**) Night Eating Questionnaire (NEQ): The prevalence of night eating syndrome (NEQ ≥ 25) was similar between groups (14.3% in PCOS vs. 11.4% in controls, *p* = 1.000), despite higher median NEQ scores in PCOS. (**D**) Chronotype and Social Jetlag: Morning chronotype was significantly less frequent in the PCOS group (5.7% vs. 28.6%), while evening chronotype was more frequent (31.4% vs. 11.4%, *p* = 0.013). (**E**) No difference was observed in the distribution of social jetlag (≥2 h vs. <2 h) between groups (*p* = 1.000).

The morningness chronotype was significantly lower in the PCOS group than in the control group, whereas the eveningness chronotype was higher than that in the control group (*p* = 0.013). The intermediate chronotype was the most common in both the control and PCOS groups. The rate of intermediate chronotypes was similar in both groups. There was no significant difference between the PCOS and control groups in terms of the number of individuals with SJL ≥ 2 h and NES. When categorized according to total test scores, the number of individuals with moderate and severe anxiety (BAI scores ≥ 16) was significantly higher in the PCOS group than in the control group (*p* = 0.030). The number of patients with depression (BDI score ≥ 17) was similar between the PCOS and control groups (*p* = 0.198) (Table 2, Figure 2).

**Figure 3:**
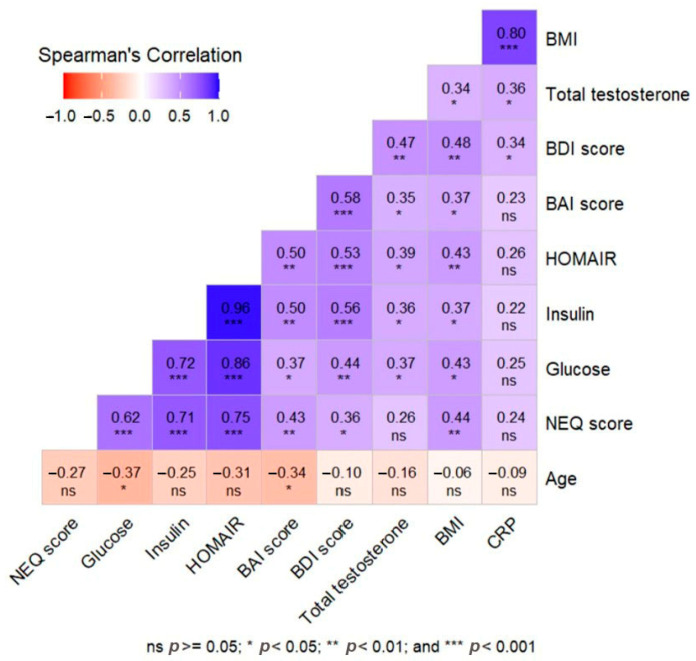
Heat map correlation matrix representation of the relationship between demographic, clinical, and laboratory data and survey scores in the PCOS group.

NEQ score showed moderate positive correlations with BAI (r = 0.432, *p* = 0.010), BDI (r = 0.360, *p* = 0.033), BMI (r = 0.436, *p* = 0.009), and FBG (r = 0.616, *p* < 0.001), while it showed strong positive correlations with insulin (r = 0.715, *p* < 0.001) and HOMA-IR (r = 0.748, *p* < 0.001). BAI score was positively correlated with BDI (r = 0.579, *p* < 0.001), BMI (r = 0.372, *p* = 0.028), FBG (r = 0.369, *p* = 0.029), insulin (r = 0.503, *p* = 0.002), HOMA-IR (r = 0.501, *p* = 0.002), and total testosterone (r = 0.345, *p* = 0.042), while it was negatively correlated with age (r = −0.343, *p* = 0.044). BDI score showed moderate positive correlation with BMI (r = 0.479, *p* = 0.004), FBG (r = 0.441, *p* = 0.008), insulin (r = 0.556, *p* < 0.001), HOMA-IR (r = 0.532, *p* < 0.001), total testosterone (r = 0.473, *p* = 0.004), and hs-CRP (r = 0.336, *p* = 0.048). The MEQ score was not significantly correlated with any of the variables (Table 3). The correlations between the demographic, clinical, and laboratory findings of patients with PCOS and the questionnaire scores are shown in the heatmap (Figure 3).

## 4. Discussion

For the first time, this cross-sectional survey study examined the relationship between PCOS and mood disorders like anxiety and depression in women, as well as circadian rhythm disorders like chronotype, social jetlag, and night eating problem. Our study’s key conclusion is that, in comparison to healthy controls without PCOS, patients with PCOS had lower MEQ scores and higher BAI, BDI, and NEQ scores than healthy controls. Studies examining the impact of PCOS on mood and circadian rhythm abnormalities are limited. In comparison to healthy controls, a recent survey study found a marked decline in sadness and anxiety indices, particularly in patients with PCOS phenotypes with hyperandrogenism [5]. In line with this, Damone et al. [30] reported that depression and anxiety were higher in women with PCOS than in healthy controls and were amplified by this and perceived stress. However, although the number of participants in these studies was sufficient, they only focused on the relationship between mood disorders and PCOS, and no comprehensive analysis was conducted on the relationship between circadian rhythms, eating disorders, and PCOS. In the current study, five different questionnaires were administered to the participants, and the relationship between PCOS and mood, circadian rhythm, and eating disorders was analyzed comprehensively.

In addition to the number of individuals with hirsutism in the PCOS group, the fact that BMI, insulin, HOMA-IR, total testosterone, DHEAS, and LH values were higher than those of the controls is important, as it is evidence that the participants in the PCOS group had disease-specific characteristics. Similarly, a high lipid profile is significant because it shows that patients with PCOS have a deteriorated metabolism. We examined the mood and circadian rhythm scores of healthy controls and PCOS patients with compromised hormonal and metabolic profiles. To compare the impact of metabolic and hormonal imbalances caused by PCOS on the survey results, it is essential that the groups include people who have received a proper diagnosis.

The high BDI and BAI scores in the PCOS group suggest that these patients are more likely to experience anxiety and despair. The increase in BAI and BDI scores in patients with PCOS may be explained by hirsutism and higher BMI values compared to healthy controls, in addition to increased androgen levels. According to a recent meta-analysis, women with PCOS had a much higher (more than six times) chance of experiencing moderate-to-severe anxiety than non-PCOS controls, which validates our hypothesis [18]. In support of this, another meta-analysis conducted by [31] reported that adolescents with PCOS exhibited more intense depression and anxiety symptoms than healthy controls [31]. The fact that individuals with PCOS exhibit more pronounced depressive symptoms in later decades suggests that PCOS-related mood changes affect individuals not only during the reproductive period but also during the perimenopausal period [32].

In addition to the metabolic and physical stress caused by PCOS, the fact that some of our participants worked in occupational groups requiring physical strength may have contributed to the increase in BAI and BDI scores as an additional stress factor. The fact that the number of patients with a BDI cut-off ≥ 17 in the PCOS group was 14 (40%) and the number of patients in the control group was 8 (22.9%) is evidence that individuals with PCOS are more prone to depression than those without. Similarly, the fact that the number of participants with a BAI cutoff ≥ 16 in the PCOS group was 20 (57.1%) and the number of participants in the control group was 10 (28.6%) indicates that PCOS stimulates perceived stress for the development of anxiety. The cohabitation of anxiety and depression is supported by the significant correlation between the BAI and BDI scores of patients with PCOS. The significant correlation between the BAI score and BMI, insulin, HOMA-IR, and testosterone levels implies that the onset of anxiety symptoms is influenced by metabolic and adiposity alterations caused by PCOS. Similarly, PCOS may be a risk factor for depressed mood disorders based on the positive correlation between BDI scores and BMI, HOMA-IR, and androgens. The positive correlation between BDI scores and hs-CRP levels may indicate that mood disorders are exacerbated by chronic inflammatory diseases in patients with PCOS.

The condition in which calorie intake occurs predominantly and intentionally at night is defined as “night eating syndrome”. Although mood disorders, such as depression and anxiety, often accompany NES [33], there are no adequate observational or survey studies on the frequency of NES in women with PCOS [34]. In the analysis conducted to test how PCOS affects eating habits, although the NEQ score was higher in the PCOS group than in the control group, we did not find any difference between the groups in terms of the number of patients with NEQ cut-off ≥ 25 (11.4% vs. 14.3%, respectively). This data may indicate that eating disorders are more pronounced in patients with PCOS than in healthy individuals, but not severe enough to be classified as NES.

The high NEQ score in the PCOS group suggests that, in addition to mood disorders such as depression and anxiety, a high BMI and metabolic changes lead to eating disorders. The fact that binge eating disorder is more prevalent in women with PCOS than in healthy individuals may provide evidence for this hypothesis [35]. The association between mood and eating disorders is supported by the positive correlation between NEQ, BAI, and BDI scores. Adiposity and metabolic abnormalities caused by PCOS are also linked to eating disorders, as seen by the significant correlations between the NEQ score and BMI, glucose, insulin, and HOMA-IR. Chronotype could be another factor contributing to the high NEQ score in the PCOS group. By altering their sleep cycles, patients with PCOS may develop a propensity for evening chronotype, which may lead to night eating behaviors. The PCOS group’s increased eveningness-type chronotype compared to the control group may indicate that the chronotype had an impact on dietary differences.

SJL, a chronic mismatch between the biological and social clocks [16], occurs in two-thirds of college-aged students [36]. Its association with pathologies such as HOMA-IR and BMI suggests an increased risk of SJL in women with PCOS [9,37]. The SJL questionnaire score, which defines the difference between workday and weekend sleep patterns, was similar between the PCOS and control groups. The similarity in the number of participants with SJL ≥ 2 h and SJL < 2 h suggests that PCOS does not affect the prevalence of SJL. SJL, which has not been addressed in previous PCOS questionnaire studies, was assessed for the first time in the present questionnaire study, and it was shown that PCOS does not increase the risk of SJL. Although some participants had similar SJL scores due to shift work, they were less aware of changes in sleep quality due to light exposure on weekdays and weekends. This is a significant factor influencing the biological clocks. However, some participants with PCOS reported SJL despite not working, suggesting that the effect of PCOS on SJL may be independent of work. Nonetheless, the variations in sleep patterns between workdays and days off in those without PCOS imply that working hours impact SJL in addition to underlying medical conditions [38]. In addition to biological clock changes, parameters such as low BMI, low income, and irregular menstrual cycles have been reported to affect sleep patterns.

According to the MEQ (Morning–Eveningness Test) results, the frequencies of ECT and MCT differed between the PCOS and control groups, while the incidence of ICT was similar in the two groups. Chronotypes play a role in determining individuals’ eating habits [39]. In patients with PCOS, the ECT was significantly higher than that in the control group (31.4% vs. 11.4%) but significantly lower than that in the MCT control group (5.7% vs. 28.6%). Although many factors, such as genetics, lifestyle, and reduced light exposure, determine chronotypes [35], there are no comprehensive data on the relationship between PCOS and chronotypes.

While individuals with MCT have good mental function [40], those with ECT experience both physical and psychological problems [41]. The fact that some patients in the PCOS group were students and others were working in different occupations may explain the higher frequency of ECT in this group. Although insulin resistance and obesity have been reported to be more prevalent in evening-type individuals and less prevalent in morning-type individuals [42], we found no association between chronotypes and metabolic or physical parameters in the present study.

Although this survey study is the first to report the association between PCOS and mood disorders, social jetlag, chronotypes, and eating disorders, it has several limitations. Although the cross-sectional design provides insight into the relationship between PCOS and survey results, it is insufficient to establish causality. Longitudinal studies with larger sample sizes are needed to clarify the effects of PCOS on mood, chronotype, and disordered eating. Including individuals with PCOS from different age groups, university students, and socioeconomic statuses may help distinguish the effects of PCOS-related variables on the survey results.

## 5. Conclusions

This experimental study examined, for the first time, the impact of PCOS on mood disorders, chronotypes, social jetlag, and eating disorders. The observation that scores on the Beck Anxiety Inventory (BAI), Beck Depression Inventory (BDI), and Night Eating Questionnaire (NEQ) surpassed those of healthy individuals within the studied cohort may signify that PCOS acts as a catalyst for mood and eating disorders. The higher prevalence of evening chronotype (ECT) individuals in the PCOS group than in the control group, alongside the higher representation of morning chronotype (MCT) individuals in the control group, provides compelling evidence that PCOS adversely influences circadian rhythm. The positive correlation between NEQ, BDI, and BAI scores and metabolic, endocrine, and adiposity parameters indicates that numerous syndrome-specific factors associated with PCOS may not only trigger mood and eating disorders but also influence chronotype. These outcomes represent a significant advancement in understanding the extensive ramifications of PCOS on non-infertility-related issues in reproductive-age populations. Given the survey findings, it is recommended that healthcare providers include an assessment of the psychological dimensions of the disorder, along with its metabolic, endocrine, and infertility-related consequences, when treating young individuals diagnosed with PCOS. Despite the limitations imposed by the small sample size and cross-sectional nature of the study, these factors are instrumental in facilitating future longitudinal studies with larger cohorts. Consequently, insightful findings regarding the impact of PCOS on mood, eating behaviors, and chronotypes have the potential to form the basis for psychotherapeutic interventions for patients with PCOS.

## Figures and Tables

**Table 1:** Demographic, clinical, and laboratory characteristics of the PCOS and control groups.

Parameter	Control (n = 35)	PCOS (n = 35)	*p*-Value
Age (years)	25.09 ± 4.78	23.40 ± 3.94	0.112 **^a^**
Body Mass Index (kg/m^2^)	22.25 ± 4.27	26.11 ± 6.38	0.004 **^a^**
Waist Circumference (cm)	77.31 ± 10.97	81.74 ± 13.44	0.136 **^a^**
Hip Circumference (cm)	101.63 ± 9.70	106.86 ± 13.47	0.067 **^a^**
Hirsutism	0 (0.0%)	20 (57.1%)	<0.001 **^c^**
BAI score	12 (2.5–16.5)	16 (11–24)	0.006 **^b^**
BDI score	9 (4–15.5)	16 (10–22)	0.007 **^b^**
NEQ score	14 (12–21)	17 (15–21.5)	0.013 **^b^**
MEQ score	52.71 ± 9.02	46.43 ± 8.88	0.005 **^a^**
Fasting plasma glucose (mg/dL)	83.88 ± 7.57	86.17 ± 5.76	0.160 **^a^**
Insulin (µU/mL)	8.20 (6.87–10.1)	11.18 (10.14–12.58)	<0.001 **^b^**
HOMAIR	1.72 (1.43–2.14)	2.38 (2.03–2.64)	<0.001 **^b^**
HDL-C (mg/dL)	60.8 ± 14.84	55.40 ± 14.17	0.125 **^a^**
LDL-C (mg/dL)	87.94 ± 23.23	123.83 ± 34.65	<0.001 **^a^**
Total cholesterol (mg/dL)	165.94 ± 35.63	202.14 ± 44.18	<0.001 **^a^**
Triglycerides (mg/dL)	72 (56–100.5)	84 (67–121.5)	0.226 **^b^**
Total testosterone (ng/dL)	26.87 ± 6.7	32.78 ± 10.32	0.006 **^a^**
DHEAS (µg/dL)	188.98 ± 41.21	253.48 ± 118.78	0.004 **^a^**
Estradiol (pg/mL)	42.2 (35.5–49)	43.61 (40.51–59.68)	0.090 **^b^**
FSH (IU/L)	4.92 ± 1.84	5.47 ± 1.70	0.195 **^a^**
LH (IU/L)	5.14 (3.67–5.76)	8.73 (4.91–12.27	<0.001 **^b^**
*Progesterone* (ng/mL)	1.10 (0.77–1.71)	1.03 (0.76–1.52)	0.796 **^b^**
Prolactin (ng/mL)	10 (8.15–13)	10.26 (7.37–15.34)	0.805 **^b^**
TSH (µIU/mL)	2.47 ± 0.38	2.43 ± 1.06	0.824 **^a^**
hs-CRP (mg/L)	1.5 (1.0–2.2)	1.84 (0.74–3.39)	0.507 **^b^**

^a^ Independent sample *t*-test, ^b^ Mann–Whitney U test, ^c^ Chi-square test. Significant *p*-values are shown in bold. BAI; Beck Anxiety Inventory, BDI; Beck Depression Inventory, DHEAS; Dehydroepiandrosterone Sulfate, FSH; Follicle-Stimulating Hormone, HDL-C; High-density lipoprotein cholesterol, HOMA-IR; Homeostatic model assessment of insulin resistance, LDL-C; Low-density lipoprotein cholesterol, LH; Luteinizing Hormone, MEQ; Morningness–Eveningness Questionnaire, NEQ; Night Eating Questionnaire, PCOS; Polycystic ovary syndrome, TSH; Thyroid-Stimulating Hormone, hs-CRP; High-sensitive C-reactive protein.

**Table 2:** Distribution of participants’ social jetlag status, chronotypes, NEQ, BAI, and BDI scores.

	Control (n, %)	PCOS (n, %)	*p*-Value
Social Jetlag status Social Jetlag ≥ 2 h Social Jetlag < 2 h	6 (17.1) 29 (82.9)	6 (17.1) 29 (82.9)	1.000 ^c^
Chronotypes Morningness Intermediate type Eveningness	10 (28.6) 21 (60) 4 (11.4)	2 (5.7) * 22 (62.9) 11 (31.4) *	0.013 ^c^
NEQ score (cut-off ≥ 25)	4 (11.4)	5 (14.3)	1.000 ^d^
BAI score (cut-off ≥ 16)	10 (28.6)	20 (57.1)	0.030 ^c^
BDI score (cut-off ≥ 17)	8 (22.9)	14 (40.0)	0.198 ^c^

^c^ Chi-square test, ^d^ Fisher’s exact test, * *p* < 0.05 vs. control group. Significant *p*-values are shown in bold. BAI; Beck Anxiety Inventory, BDI; Beck Depression Inventory, NEQ; Night Eating Questionnaire, PCOS; Polycystic ovary syndrome.

**Table 3:** Correlation of survey scores with the demographic, laboratory, and clinical findings of patients with PCOS.

	NEQ		MEQ		BAI		BDI	
	r	*p*-Value	r	*p*-Value	r	*p*-Value	r	*p*-Value
MEQ	−0.188	0.279	**-**	**-**	**-**	**-**	**-**	**-**
BAI	**0.432**	**0.010**	0.044	0.803	**-**	**-**	**-**	**-**
BDI	**0.360**	**0.033**	−0.002	0.993	**0.579**	**<0.001**	**-**	**-**
Age (years)	−0.268	0.119	0.202	0.246	**−0.343**	**0.044**	−0.099	0.570
BMI (kg/m^2^)	**0.436**	**0.009**	−0.005	0.979	**0.372**	**0.028**	**0.479**	**0.004**
Fasting plasma glucose (mg/dL)	**0.616**	**<0.001**	0.008	0.963	**0.369**	**0.029**	**0.441**	**0.008**
Insulin (µU/mL)	**0.715**	**<0.001**	−0.072	0.680	**0.503**	**0.002**	**0.556**	**<0.001**
HOMAIR	**0.748**	**<0.001**	−0.016	0.927	**0.501**	**0.002**	**0.532**	**<0.001**
HDL (mg/dL)	−0.124	0.479	−0.118	0.499	−0.286	0.096	−0.146	0.401
LDL (mg/dL)	−0.058	0.743	0.152	0.383	0.113	0.517	0.180	0.302
Total cholesterol (mg/dL)	−0.079	0.653	0.126	0.469	0.064	0.717	0.234	0.177
Triglyceride (mg/dL)	0.038	0.830	0.140	0.424	0.255	0.139	0.212	0.221
Total testosterone (ng/dL)	0.255	0.139	0.117	0.503	**0.345**	**0.042**	**0.473**	**0.004**
DHEAS (µg/dL)	−0.065	0.710	−0.010	0.953	−0.013	0.940	0.146	0.402
Estradiol (pg/mL)	0.087	0.618	−0.061	0.728	0.050	0.777	0.038	0.830
FSH (IU/L)	−0.215	0.215	0.308	0.072	−0.252	0.144	−0.256	0.138
LH (IU/L)	−0.204	0.240	0.177	0.310	0.114	0.514	0.192	0.268
Progesterone (ng/mL)	−0.033	0.851	−0.018	0.920	0.053	0.763	−0.025	0.885
Prolactin (ng/mL)	−0.185	0.287	−0.139	0.426	−0.073	0.676	0.034	0.848
TSH (µIU/mL)	0.055	0.752	−0.045	0.796	0.245	0.156	0.171	0.326
hs-CRP (mg/L)	0.243	0.159	−0.082	0.641	0.233	0.178	**0.336**	**0.048**

Significant results are shown in bold. BAI; Beck Anxiety Inventory, BDI; Beck Depression Inventory, DHEAS; Dehydroepiandrosterone Sulfate, FSH; Follicle-Stimulating Hormone, HDL-C; High-density lipoprotein cholesterol, HOMA-IR; Homeostatic model assessment of insulin resistance, LDL-C; Low-density lipoprotein cholesterol, LH; Luteinizing Hormone, MEQ; Morningness–Eveningness Questionnaire, NEQ; Night Eating Questionnaire, TSH; Thyroid-Stimulating Hormone, hs-CRP; High-sensitive C-reactive protein.

## Data Availability

All data supporting the results of this study are available from the corresponding author upon reasonable request.

## References

[1] Shahid R., Iahtisham Ul H., Mahnoor, Awan K.A., Iqbal M.J., Munir H., Saeed I. (2022). Diet and lifestyle modifications for effective management of polycystic ovarian syndrome (PCOS). J. Food Biochem..

[2] Szczuko M., Kikut J., Szczuko U., Szydlowska I., Nawrocka-Rutkowska J., Zietek M., Verbanac D., Saso L. (2021). Nutrition Strategy and Life Style in Polycystic Ovary Syndrome-Narrative Review. Nutrients.

[3] Holton S., Hammarberg K., Johnson L. (2018). Fertility concerns and related information needs and preferences of women with PCOS. Hum. Reprod. Open.

[4] Teede H.J., Tay C.T., Laven J., Dokras A., Moran L.J., Piltonen T.T., Costello M.F., Boivin J., Redman L.M., Boyle J.A. (2023). Recommendations from the 2023 International Evidence-based Guideline for the Assessment and Management of Polycystic Ovary Syndromedagger. Hum. Reprod..

[5] Cetinkaya Altuntas S., Celik O., Ozer U., Colak S. (2022). Depression, anxiety, body image scores, and sexual dysfunction in patients with polycystic ovary syndrome according to phenotypes. Gynecol. Endocrinol..

[6] Vatier C., Christin-Maitre S. (2024). Epigenetic/circadian clocks and PCOS. Hum. Reprod..

[7] Gungor N.D., Celik O., Ulug U., Celik N., Ersahin A., Gungor K., Yurci A., Yardim M., Kobaner M., Tektemur A. (2024). Hyperandrogenemia impairs endometrial vitamin D receptor expression in polycystic ovary syndrome. Gynecol. Endocrinol..

[8] Fischer D., Lombardi D.A., Marucci-Wellman H., Roenneberg T. (2017). Chronotypes in the US—Influence of age and sex. PLoS ONE.

[9] Parkar S.G., Kalsbeek A., Cheeseman J.F. (2019). Potential Role for the Gut Microbiota in Modulating Host Circadian Rhythms and Metabolic Health. Microorganisms.

[10] Vetter C. (2020). Circadian disruption: What do we actually mean?. Eur. J. Neurosci..

[11] Rea M.S., Bierman A., Figueiro M.G., Bullough J.D. (2008). A new approach to understanding the impact of circadian disruption on human health. J. Circadian Rhythm..

[12] Inokawa H., Umemura Y., Shimba A., Kawakami E., Koike N., Tsuchiya Y., Ohashi M., Minami Y., Cui G., Asahi T. (2020). Chronic circadian misalignment accelerates immune senescence and abbreviates lifespan in mice. Sci. Rep..

[13] Morris C.J., Purvis T.E., Mistretta J., Hu K., Scheer F. (2017). Circadian Misalignment Increases C-Reactive Protein and Blood Pressure in Chronic Shift Workers. J. Biol. Rhythm..

[14] Kantermann T., Eastman C.I. (2018). Circadian phase, circadian period and chronotype are reproducible over months. Chronobiol. Int..

[15] Chellappa S.L., Morris C.J., Scheer F. (2019). Effects of circadian misalignment on cognition in chronic shift workers. Sci. Rep..

[16] Wittmann M., Dinich J., Merrow M., Roenneberg T. (2006). Social jetlag: Misalignment of biological and social time. Chronobiol. Int..

[17] Pagani L., Semenova E.A., Moriggi E., Revell V.L., Hack L.M., Lockley S.W., Arendt J., Skene D.J., Meier F., Izakovic J. (2010). The physiological period length of the human circadian clock in vivo is directly proportional to period in human fibroblasts. PLoS ONE.

[18] Cooney L.G., Lee I., Sammel M.D., Dokras A. (2017). High prevalence of moderate and severe depressive and anxiety symptoms in polycystic ovary syndrome: A systematic review and meta-analysis. Hum. Reprod..

[19] Senturk S., Kagitci M., Baltacioglu M., Delibas D.D., Ustuner I. (2025). The influence of primary dysmenorrhea on chronotypes, social jetlag, and night eating habits: A cross-sectional study of anxiety and depression. BMC Women’s Health.

[20] Muscogiuri G., Barrea L., Aprano S., Framondi L., Di Matteo R., Laudisio D., Pugliese G., Savastano S., Colao A., on behalf of the Opera Prevention Project (2020). Chronotype and Adherence to the Mediterranean Diet in Obesity: Results from the Opera Prevention Project. Nutrients.

[21] The Rotterdam ESHRE/ASRM-Sponsored PCOS Consensus Workshop Group (2004). Revised 2003 consensus on diagnostic criteria and long-term health risks related to polycystic ovary syndrome. Fertil. Steril..

[22] Matthews D.R., Hosker J.P., Rudenski A.S., Naylor B.A., Treacher D.F., Turner R.C. (1985). Homeostasis model assessment: Insulin resistance and beta-cell function from fasting plasma glucose and insulin concentrations in man. Diabetologia.

[23] Horne J.A., Ostberg O. (1976). A self-assessment questionnaire to determine morningness-eveningness in human circadian rhythms. Int. J. Chronobiol..

[24] Punduk Z., Gur H., Ercan I. (2005). A reliability study of the Turkish version of the mornings-evenings questionnaire. Turk. Psikiyatr. Derg..

[25] Allison K.C., Lundgren J.D., O’Reardon J.P., Martino N.S., Sarwer D.B., Wadden T.A., Crosby R.D., Engel S.G., Stunkard A.J. (2008). The Night Eating Questionnaire (NEQ): Psychometric properties of a measure of severity of the Night Eating Syndrome. Eat. Behav..

[26] Atasoy N., Saracli O., Konuk N., Ankaralı H., Guriz S.O., Akdemir A., Sevincer G.M., Atik L. (2014). The reliability and validity of Turkish version of the Night Eating Questionnaire in psychiatric outpatient population. Anatol. J. Psychiatry.

[27] Hisli N. (1988). A study on the validity of Beck Depression Inventory. J. Psychol..

[28] Ulusoy M., Sahin N.H., Erkmen H. (1998). Turkish Version of the Beck Anxiety Inventory: Psychometric Properties. J. Cogn. Psychother..

[29] Olivoto T., Lucio A.D. (2020). Metan: An R package for multi-environmenttrial analysis. Methods Ecol. Evol..

[30] Damone A.L., Joham A.E., Loxton D., Earnest A., Teede H.J., Moran L.J. (2019). Depression, anxiety and perceived stress in women with and without PCOS: A community-based study. Psychol. Med..

[31] Nasiri-Amiri F., Faramarzi M., Omidvar S., Alizadeh-Navaei R. (2023). Depression and anxiety in adolescents and young women with polycystic ovary syndrome: A systematic review and meta-analysis. Int. J. Adolesc. Med. Health.

[32] Greenwood E.A., Yaffe K., Wellons M.F., Cedars M.I., Huddleston H.G. (2019). Depression Over the Lifespan in a Population-Based Cohort of Women with Polycystic Ovary Syndrome: Longitudinal Analysis. J. Clin. Endocrinol. Metab..

[33] Guentcheva I., Dugas E.N., Hanusaik N., Drapeau V., Sylvestre M.P., O’Loughlin J. (2020). Depression symptoms and night eating in young adulthood. Eat. Weight. Disord..

[34] Lee I., Cooney L.G., Saini S., Smith M.E., Sammel M.D., Allison K.C., Dokras A. (2017). Increased risk of disordered eating in polycystic ovary syndrome. Fertil. Steril..

[35] Asdaq S.M.B., Jomah S., Hasan R., Al-Baroudi D., Alharbi M., Alsubaie S., Buhamad M.H., Alyahya B., Al-Yamani M.J. (2020). Impact of polycystic ovary syndrome on eating behavior, depression and health related quality of life: A cross-sectional study in Riyadh. Saudi J. Biol. Sci..

[36] Roenneberg T., Wirz-Justice A., Merrow M. (2003). Life between clocks: Daily temporal patterns of human chronotypes. J. Biol. Rhythm..

[37] Koopman A.D., Rauh S.P., Riet E.V.T., Groeneveld L., Van Der Heijden A.A., Elders P.J., Dekker J.M., Nijpels G., Beulens J.W., Rutters F. (2017). The Association between Social Jetlag, the Metabolic Syndrome, and Type 2 Diabetes Mellitus in the General Population: The New Hoorn Study. J. Biol. Rhythm..

[38] Hu Z., Tang L., Chen L., Kaminga A.C., Xu H. (2020). Prevalence and Risk Factors Associated with Primary Dysmenorrhea among Chinese Female University Students: A Cross-sectional Study. J. Pediatr. Adolesc. Gynecol..

[39] Naja F., Hasan H., Khadem S.H., Buanq M.A., Al-Mulla H.K., Aljassmi A.K., Faris M.E. (2021). Adherence to the Mediterranean Diet and Its Association with Sleep Quality and Chronotype Among Youth: A Cross-Sectional Study. Front. Nutr..

[40] Diaz-Morales J.F., Jankowski K.S., Vollmer C., Randler C. (2013). Morningness and life satisfaction: Further evidence from Spain. Chronobiol. Int..

[41] Caliandro R., Streng A.A., van Kerkhof L.W.M., van der Horst G.T.J., Chaves I. (2021). Social Jetlag and Related Risks for Human Health: A Timely Review. Nutrients.

[42] Rawat A., Gangwar A.K., Tiwari S., Kant S., Garg R.K., Singh P.K. (2019). Sleep quality and insulin resistance in adolescent subjects with different circadian preference: A cross-sectional study. J. Family Med. Prim. Care.

